# Naïve HIV late presenters – a study for 35 months in the Infectious Diseases Hospital Iaşi, Romania

**DOI:** 10.1186/1471-2334-14-S4-P1

**Published:** 2014-05-29

**Authors:** Carmen Manciuc, Maria Alexandra Largu, Andrei Vâță, Cristina Nicolau, Liviu Jany Prisăcariu, Carmen Dorobăț

**Affiliations:** 1 Infectious Diseases Hospital “Sf Parascheva”, Iaşi, Romania

## 

The HIV/AIDS Regional Center in Iaşi follows approximately 1460 HIV-positive persons from 6 counties in the Moldova region of Romania. Objectives: The study aims to evaluate the number of late presenters in January 2011-November 2013.

We evaluated patients admitted to the Infectious Diseases Hospital in Iaşi, for a period of 35 months, from a virological and immunological point of view. We considered as “late presenters” naïve patients with a CD4 cell count of less than 200/cmm.

From January 2011 to November 2013 there were 143 naïve patients hospitalized, of which 60 (42%) were considered late presenters, with a CD4 cell count of less than 200/cmm and a viral load of more than 10,000 copies/mL; 48 patients (80%) had a CD4<50/cmm; 14 patients died in the first 4 weeks of positive diagnosis, that is 23.3% of late presenters and 10% of all naïve patients. The median age for the late presenters was 28.4 years. However 6 patients came from the “Romanian pediatric cohort” (infected at a young age, in the early 1990’s), and were considered “slow-progressors”, living with an undetected infection for 20 years. The clinical spectrum on diagnosis was: 3 cases of pregnant women detected during routine pregnancy tests; 8 patients from the men who have sex with men (MSM) population; 4 cases of pneumocystosis; 10 cases of tuberculosis; 6 patients from serodiscordant couples (infected by their HIV-positive partners); 4 patients came from other clinical services – neurosurgery, pneumology etc. We initiated pneumocystosis and TB therapy in the necessary cases, and antiretroviral therapy.

In the N-E region of our country, we still report a high number of late presenters (42% of all naive patients), with a high mortality rate (10%). The fact that HIV-positive patients were identified in other services than the Infectious Diseases department shows a much improved collaboration between medical specialties. However, campaigns to raise awareness about the sexually transmitted diseases in vulnerable populations are still necessary.

